# A Case of Early PreserFlo MicroShunt Obstruction Following Presumed Inadvertent Ciliary Sulcus Implantation

**DOI:** 10.7759/cureus.116821

**Published:** 2026-09-24

**Authors:** Yuki Takeda, Haruka Ayama, Sachiko Kaidzu, Masanari Ichinose, Hinako Ohtani, Chisako Ida, Masaki Tanito

**Affiliations:** 1 Department of Ophthalmology, Shimane University Faculty of Medicine, Izumo, JPN

**Keywords:** case report, ciliary sulcus, preserflo microshunt, primary open-angle glaucoma, tube obstruction, uveal tissue

## Abstract

We report a case of early postoperative PreserFlo MicroShunt (PFM; Santen Pharmaceutical Co., Ltd., Osaka, Japan) obstruction following presumed inadvertent ciliary sulcus implantation. A 67-year-old Japanese man with primary open-angle glaucoma underwent superonasal PFM implantation in the right eye, which had previously undergone cataract surgery, scleral buckling, and microhook ab interno trabeculotomy. The preoperative intraocular pressure (IOP) in the right eye was 26 mmHg on two classes of topical IOP-lowering medications. Although the proximal tip was not visible in the anterior chamber, aqueous humor outflow was confirmed intraoperatively, and the device was left in place. On postoperative day 1, IOP was 24 mmHg, and no filtering bleb was observed. The PFM was removed, and a new device was implanted into the anterior chamber through an adjacent scleral tunnel. The IOP was 6 mmHg on postoperative day 1 after the second PFM implantation. Stereomicroscopic examination of the first explanted PFM device revealed intraluminal pigmented tissue fragments consistent with uveal tissue, although their precise origin could not be established. Bleb needling was performed at five weeks because the IOP had increased to 31 mmHg, presumably due to bleb encapsulation. The IOP was 15 mmHg at three months after reoperation. This instructive case illustrates the importance of confirming the scleral tract trajectory and implant position, as apparently satisfactory intraoperative outflow does not ensure sustained tube patency. These findings should not be generalized to the safety or efficacy of intentional ciliary sulcus implantation.

## Introduction

The PreserFlo MicroShunt (PFM; Santen Pharmaceutical Co., Ltd., Osaka, Japan) is a minimally invasive bleb surgery device that lowers intraocular pressure (IOP) by diverting aqueous humor from the anterior chamber to the subconjunctival space. Compared with trabeculectomy, PFM implantation provides less pronounced IOP reduction but may offer advantages in certain aspects of postoperative safety [1,2] and induces less astigmatism [3]. Effective IOP reduction has also been reported in Japanese patients with inadequately controlled glaucoma despite previous glaucoma surgery [4]. Although the PFM is typically implanted in a superior quadrant with its proximal tip positioned in the anterior chamber, alternative approaches, including inferior quadrant placement [5] and ciliary sulcus insertion [6,7], have been described.

Corneal endothelial cell loss remains a concern following anterior chamber implantation of the PFM, and eyes with exfoliation glaucoma may be particularly vulnerable [8-10]. Ciliary sulcus placement has therefore been proposed as an alternative for eyes at increased risk of endothelial damage [6,7]. However, the available evidence on this approach remains limited, consisting primarily of case reports [6,7]. The surgical technique has not been standardized, and the technical considerations and complications specific to sulcus placement have yet to be fully characterized.

Herein, we report a case of presumed inadvertent ciliary sulcus implantation of the PFM in a patient with primary open-angle glaucoma (POAG). Although aqueous humor flow through the device was initially confirmed intraoperatively, early postoperative filtration failure occurred because of tube obstruction by pigmented tissue consistent with uveal tissue. We describe the clinical course, intraoperative findings during revision surgery, and microscopic characteristics of the obstructing material to highlight potential pitfalls associated with unintended placement of the PFM posterior to the iris.

## Case presentation

A 67-year-old Japanese man presented with POAG in both eyes. He had undergone surgery for retinal detachment in the right eye in his late 20s. He subsequently underwent phacoemulsification with intraocular lens (IOL) implantation in the right eye and extracapsular cataract extraction with IOL implantation in the left eye. Topical glaucoma treatment was later initiated in both eyes, although the exact timing was unknown. His systemic medical history was notable for asthma, with no other significant medical conditions.

At the initial examination at our hospital, the IOP measured by Goldmann applanation tonometry was 21 mmHg in the right eye and 23 mmHg in the left eye while the patient was receiving four classes of topical IOP-lowering medications (bimatoprost, brinzolamide, brimonidine, and ripasudil) and oral acetazolamide. Gonioscopy revealed open angles in both eyes. The IOLs were well positioned, and the vertical cup-to-disc ratio was 0.9 bilaterally. Conjunctival scarring associated with a segmental scleral buckle was present in the superotemporal quadrant of the right eye, whereas scarring from previous cataract surgery was present superiorly in the left eye. Humphrey Field Analyzer (HFA; Carl Zeiss Meditec, Dublin, CA, USA) testing was performed using both the 30-2 and 10-2 programs and showed paracentral scotomas in both eyes. The mean deviation (MD) values on the 30-2 program were -8.81 dB in the right eye and -7.01 dB in the left eye, whereas those on the 10-2 program were -11.49 dB and -21.10 dB, respectively. Corneal endothelial cell density (CECD) was 2,576 cells/mm² in the right eye and 2,467 cells/mm² in the left eye, and central corneal thickness (CCT) was 530 μm and 535 μm, respectively (EM-3000; Tomey Corporation, Nagoya, Japan). Anterior chamber flare values were 13.7 photons/ms in the right eye and 0.9 photons/ms in the left eye (FM-600α; Kowa, Tokyo, Japan). The patient subsequently underwent 25-gauge pars plana vitrectomy combined with Ahmed Glaucoma Valve FP7 implantation in the left eye and microhook ab interno trabeculotomy in the right eye. Three years after trabeculotomy, IOP in the right eye increased to 26 mmHg despite treatment with two classes of topical IOP-lowering medications. The visual field showed progression on the HFA 30-2 program, with an MD of -11.31 dB, while the MD on the 10-2 program was -18.80 dB. Therefore, PFM implantation was planned to achieve further IOP reduction.

**Figure 1:**
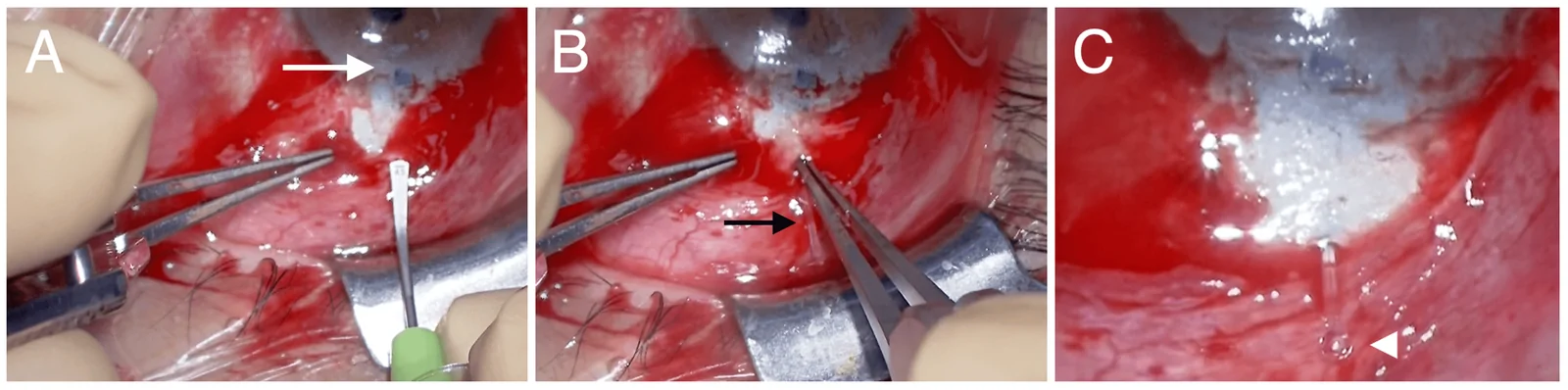
Intraoperative findings during initial PFM implantation in the right eye. (A) Creation of a scleral tunnel using a double-step knife. The knife tip is not visible in the anterior chamber (white arrow). (B) Insertion of the PFM (arrow) through the scleral tunnel. (C) Aqueous humor outflow (arrowhead) is observed from the distal end of the PFM, although the proximal tip is not visible in the anterior chamber. PFM, PreserFlo MicroShunt

**Figure 2:**
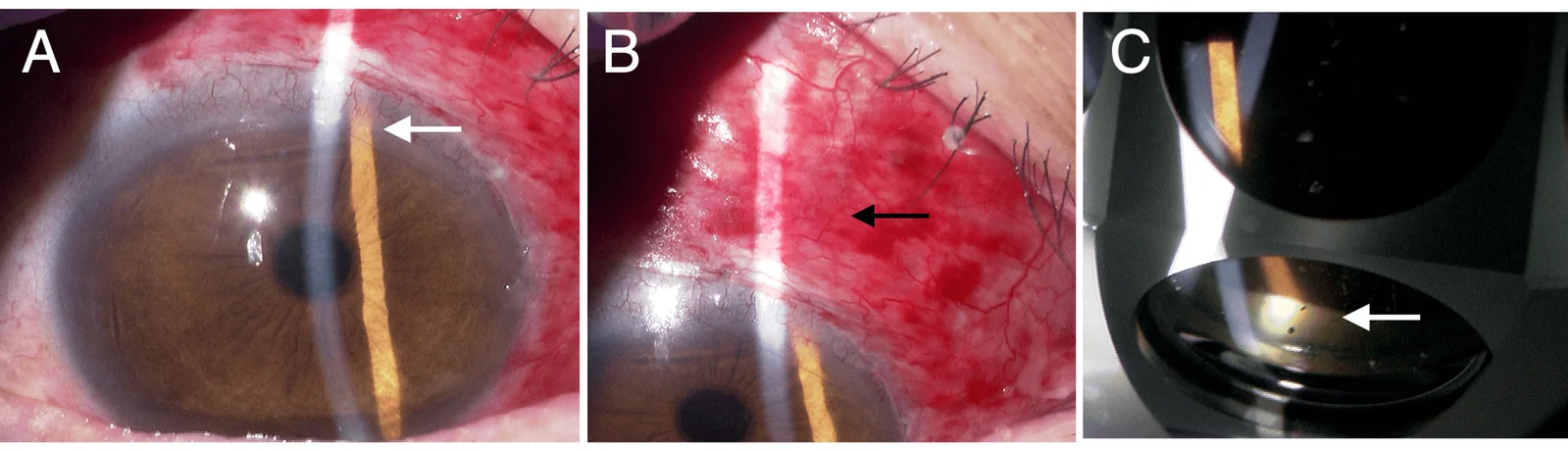
Slit-lamp and gonioscopic findings on postoperative day 1 after initial PFM implantation in the right eye. (A) The proximal tip of the PFM is not visible in the anterior chamber (white arrow). IOP is 24 mmHg. (B) No filtering bleb is observed (arrow). (C) Gonioscopy shows no visible PFM tip in the anterior chamber angle (white arrow). IOP, intraocular pressure; PFM, PreserFlo MicroShunt

PFM implantation was performed in the superonasal quadrant of the right eye, where the conjunctiva was free of scarring from the previous retinal detachment surgery (Video 1, Figure 1). After conjunctival incision, a scleral tunnel was created using the supplied double-step knife (Figure 1A), and the PFM was inserted through the tunnel (Figure 1B). Although the proximal tip could not be clearly visualized in the anterior chamber, aqueous humor outflow from the distal end was observed (Figure 1C), and the device was left in place. On postoperative day 1, the anterior chamber was deep, and IOP was 24 mmHg. Slit-lamp examination showed that the proximal tip was not visible in the anterior chamber (Figure 2A) and that no filtering bleb had formed (Figure 2B). The tip was also not visible on gonioscopy (Figure 2C). These findings raised suspicion of inadvertent ciliary sulcus placement with tube obstruction, and reoperation was performed that day.

**Video 1:** Initial PFM implantation in the right eye. PFM, PreserFlo MicroShunt

**Figure 3:**
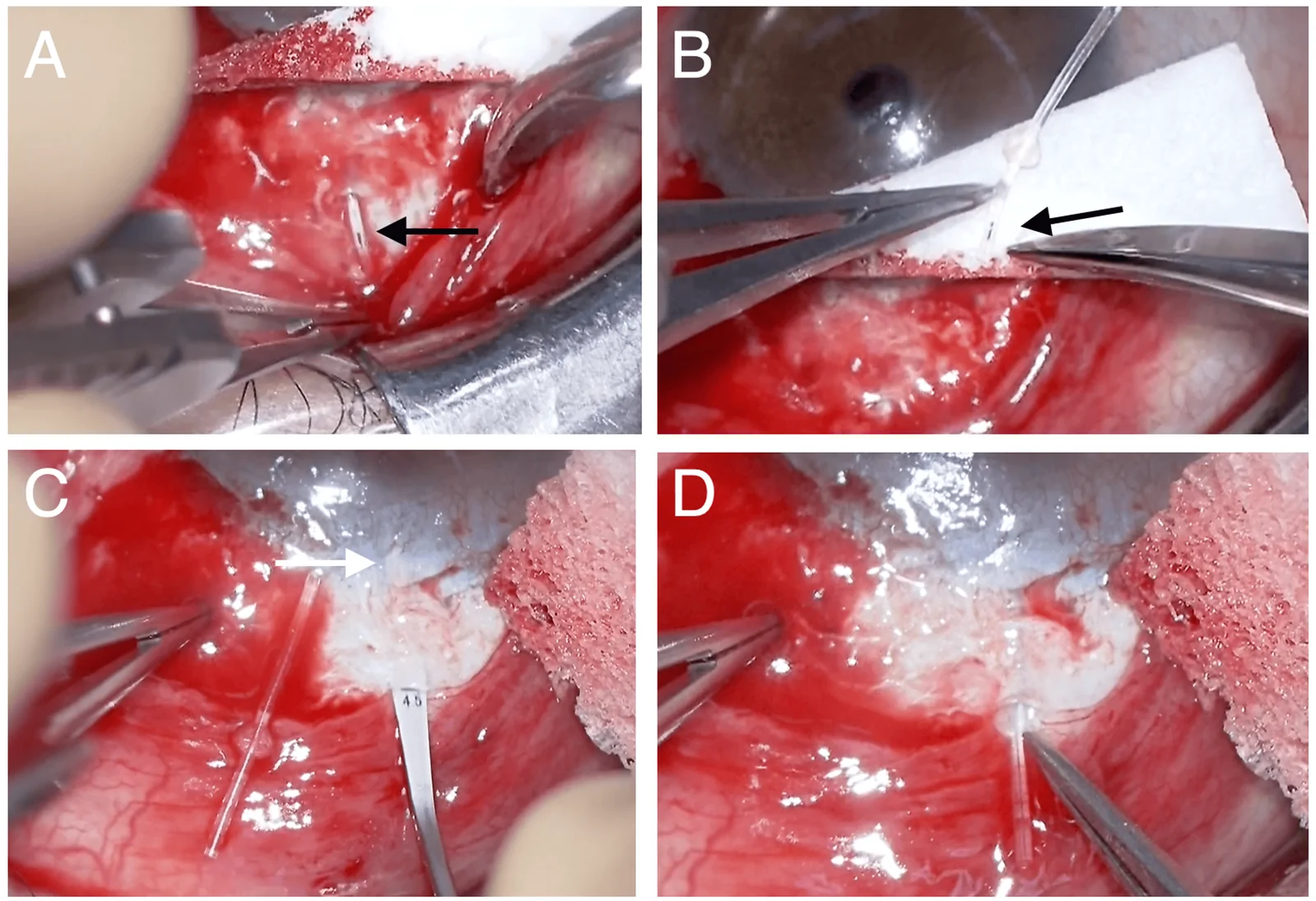
Intraoperative findings during removal of the original PFM and implantation of a new device in the right eye on postoperative day 1. (A, B) Brownish-black material (arrows) is observed within the lumen of the original PFM. (C) Creation of a new scleral tunnel using a double-step knife. The knife tip is visible in the anterior chamber (white arrow). (D) Insertion of a new PFM through the newly created tunnel. PFM, PreserFlo MicroShunt

**Figure 4:**
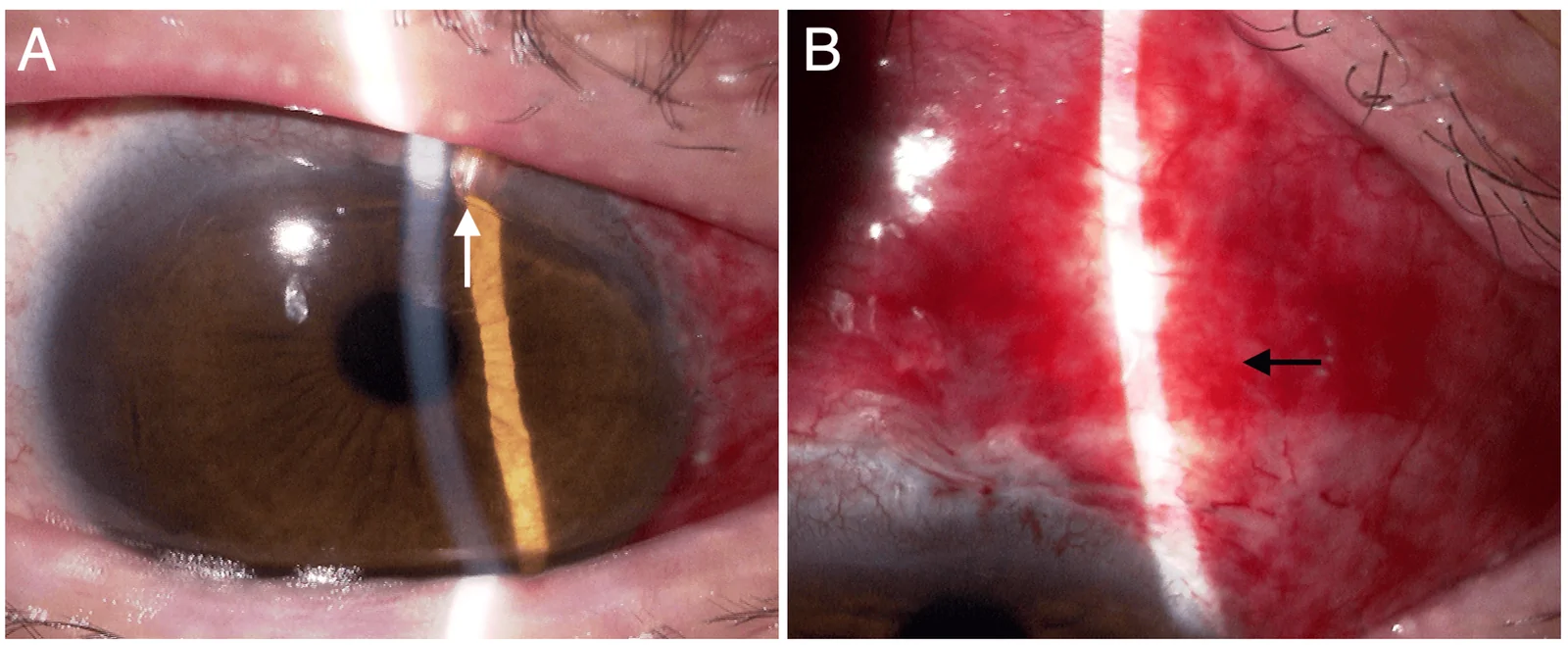
Slit-lamp findings one day after implantation of the new PFM in the right eye. (A) The proximal tip of the new PFM is visible in the anterior chamber (white arrow). IOP is 6 mmHg. (B) A well-formed filtering bleb is observed (black arrow). IOP, intraocular pressure; PFM, PreserFlo MicroShunt

The findings during reoperation are shown in Video 2 and Figure 3. The conjunctiva was reopened at the original implantation site. Examination of the PFM revealed brownish-black material obstructing the tube lumen (Figure 3A, 3B, arrows). The original device was removed, and a new scleral tunnel was created at an adjacent site using the double-step knife (Figure 3C). A new PFM was then implanted into the anterior chamber through this tunnel (Figure 3D). On the first day after reoperation, IOP was 6 mmHg. Slit-lamp examination confirmed that the proximal tip of the new device was visible in the anterior chamber (Figure 4A) and that a filtering bleb had formed (Figure 4B, black arrow). Postoperatively, topical 1.5% levofloxacin was administered four times daily for one month, and topical 0.1% betamethasone was administered four times daily for two months.

**Video 2:** Removal of the original PFM and implantation of a new device in the right eye on postoperative day 1. PFM, PreserFlo MicroShunt

**Figure 5:**
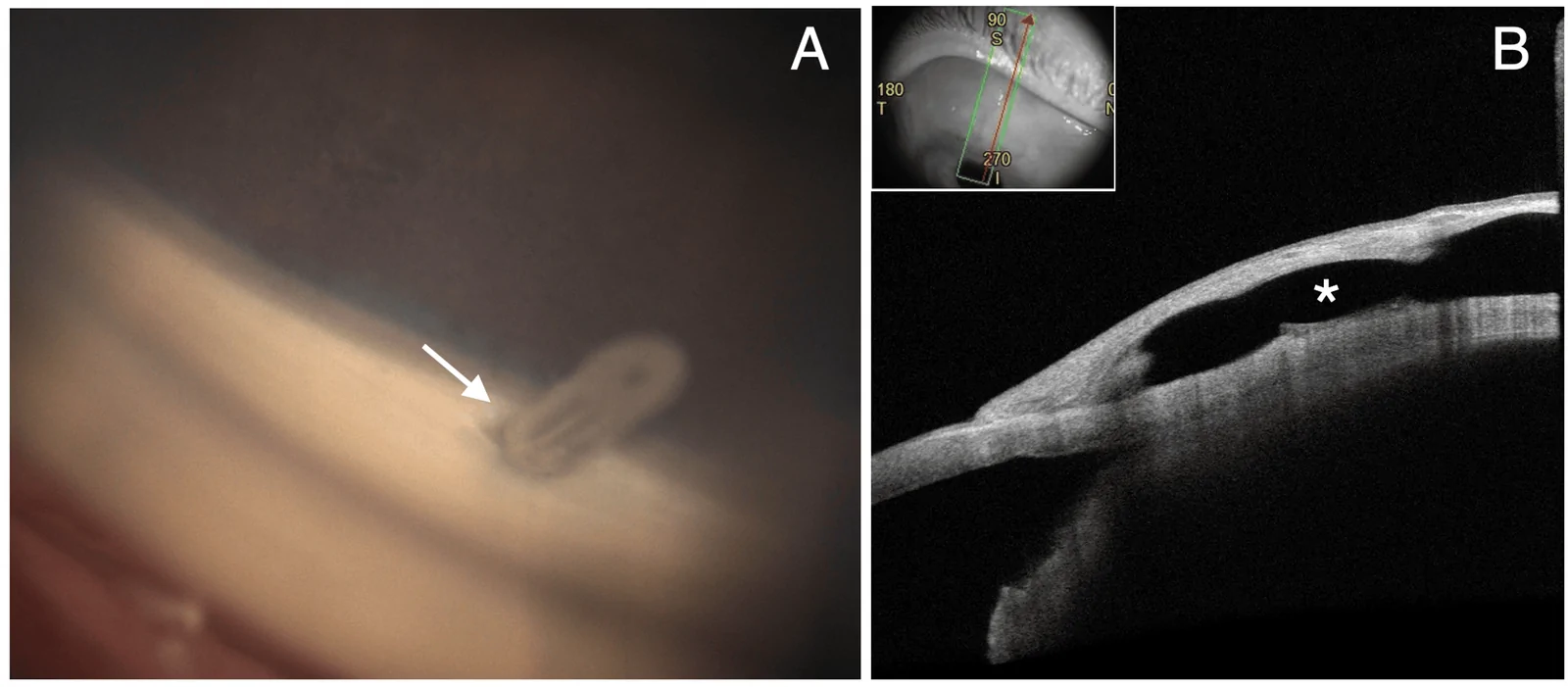
Gonioscopic and anterior segment optical coherence tomography findings five weeks after implantation of the new PFM in the right eye. (A) Gonioscopy shows the PFM entering the anterior chamber at the level of the trabecular meshwork (white arrow). (B) Anterior segment optical coherence tomography shows subconjunctival fluid accumulation (asterisk). The inset indicates the scan direction. PFM, PreserFlo MicroShunt

**Figure 6:**
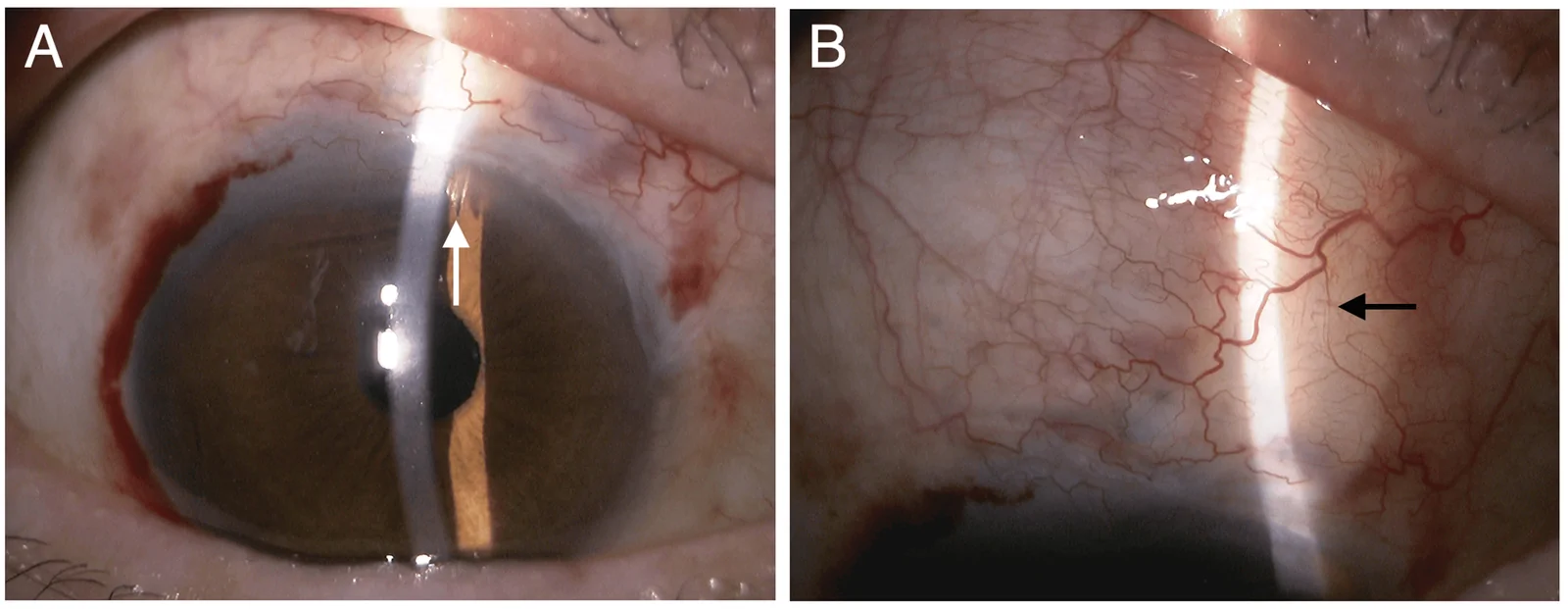
Slit-lamp findings three months after implantation of the new PFM in the right eye. (A) The position of the proximal PFM tip (white arrow) remains unchanged from that observed immediately after implantation of the new device. (B) A well-formed filtering bleb is observed (black arrow). PFM, PreserFlo MicroShunt

At five weeks after reoperation, gonioscopy showed that the PFM entered the anterior chamber at the level of the trabecular meshwork (Figure 5A). Anterior segment optical coherence tomography (CASIA2, Tomey Corporation, Nagoya, Japan) demonstrated subconjunctival fluid accumulation (Figure 5B). However, IOP had increased to 31 mmHg, presumably because of bleb encapsulation, and bleb needling was performed. At three months after reoperation, IOP improved to 15 mmHg. The position of the PFM remained unchanged (Figure 6A), and a well-formed filtering bleb was observed (Figure 6B). CECD was 2,376 cells/mm², CCT was 522 μm, and the anterior chamber flare value was 13.4 photons/ms.

**Figure 7:**
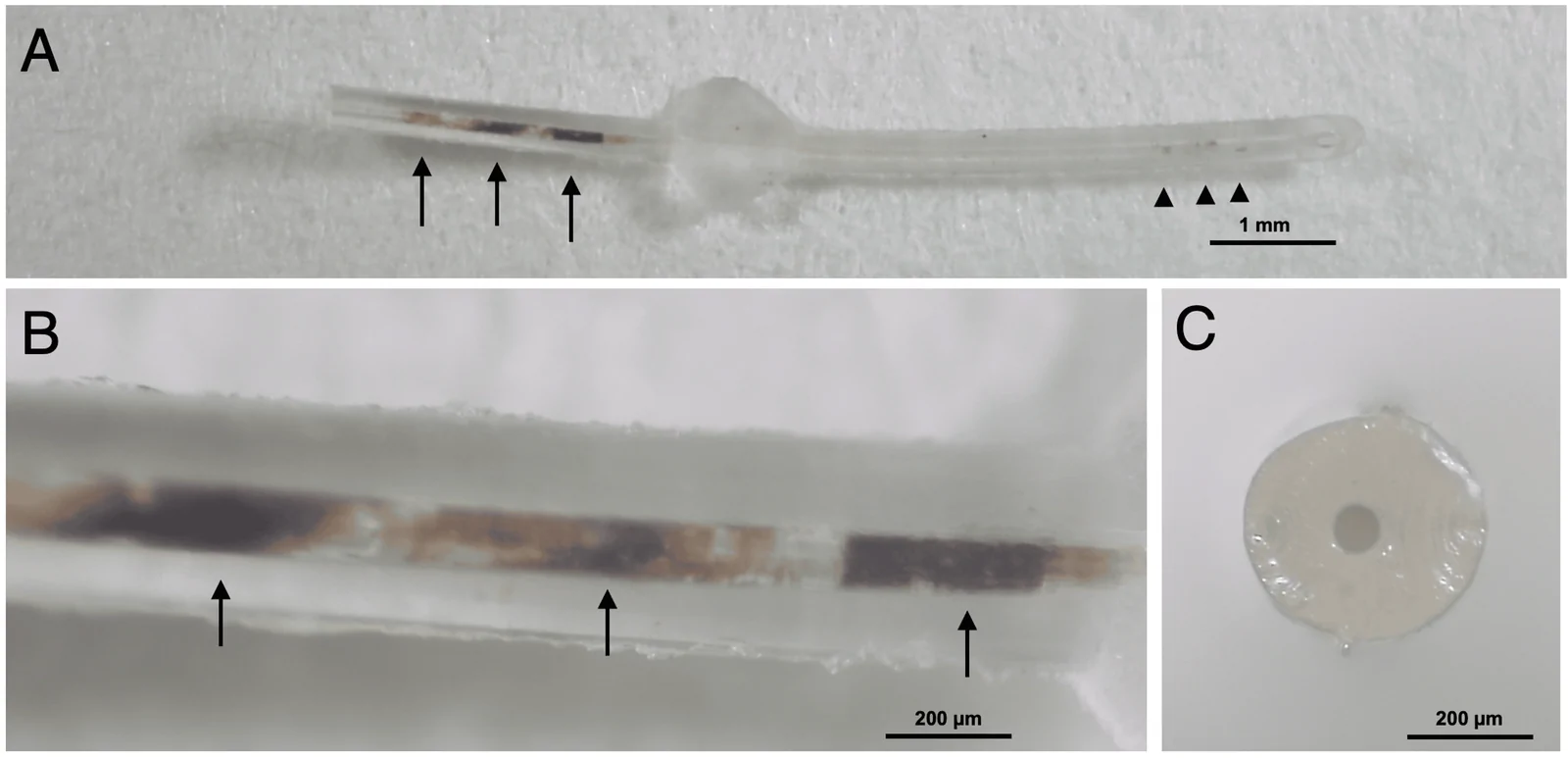
Stereomicroscopic examination of the explanted PFM. (A) Overview of the device. Brownish-black material is present within the lumen in both the distal (black arrows) and proximal (black arrowheads) portions. (B) Higher-magnification view of the obstructing material in the distal portion of the device (arrows). (C) Cross-sectional view of the PFM tube. The lumen, which has a nominal internal diameter of 70 μm, shows no collapse or deformation. PFM, PreserFlo MicroShunt

**Figure 8:**
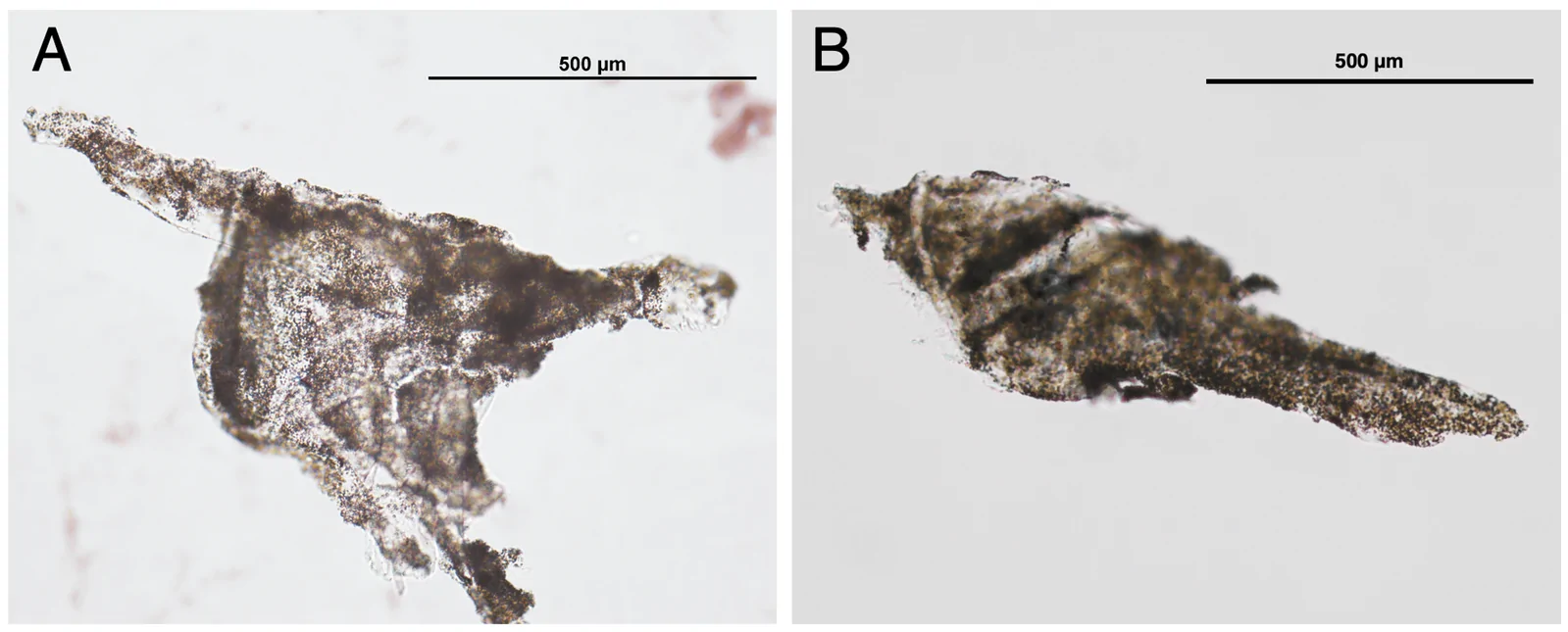
Stereomicroscopic examination of tissue fragments recovered from the PFM lumen. (A, B) Two brownish tissue fragments recovered from the distal portion of the lumen. The fragments contain abundant melanin granules. PFM, PreserFlo MicroShunt

To investigate the cause of filtration failure after the initial implantation, the explanted PFM was examined under a stereomicroscope (Figure 7). Multiple brownish-black fragments were observed within the lumen in both the distal (Figure 7A, arrows) and proximal (Figure 7A, arrowheads) portions of the device. Relatively large fragments were particularly evident in the distal portion (Figure 7B, arrows). Cross-sectional examination showed no evidence of collapse or deformation of the tube lumen (Figure 7C). The obstructing material was recovered from the distal lumen for further stereomicroscopic examination and consisted of tissue fragments containing abundant melanin granules (Figure 8A, 8B). No blood, fibrin, or lens fragments were identified. Based on their appearance and the presumed position of the original implant, the fragments were considered consistent with uveal tissue, possibly originating from the iris or ciliary body.

## Discussion

We report a case of presumed inadvertent ciliary sulcus implantation of the PFM in an eye with POAG and a history of cataract surgery, scleral buckling for retinal detachment, and microhook ab interno trabeculotomy. During superonasal implantation, the scleral tract appeared to have been unintentionally directed posterior to the iris. Although aqueous humor outflow was confirmed intraoperatively, inadequate filtration was evident on postoperative day 1, necessitating removal of the original PFM and implantation of a new device into the anterior chamber. Examination of the explanted device revealed intraluminal pigmented tissue consistent with uveal tissue. This instructive case illustrates several technical considerations regarding confirmation of implant position and the interpretation of intraoperative flow.

The scleral tunnel was created using the dedicated double-step knife. The surgeon used the marker located 4.5 mm from the knife tip to estimate insertion depth. However, this marker provides a reference for depth rather than anatomical location and does not establish whether the knife has entered the anterior chamber or passed posterior to the iris. Direct visualization of the knife tip within the anterior chamber would therefore have been important for confirming the intended trajectory. Peripheral corneal opacity, particularly in older patients, may limit visualization. When the knife tip cannot be identified, careful probing of the wound tract with a blunt cannula or another suitable instrument may help determine the direction and extent of the tract, although the usefulness of this approach was not evaluated in the present case.

During surgery, the surgeon suspected unintended placement when the proximal tip of the PFM could not be visualized in the anterior chamber. Nevertheless, the device was left in place because aqueous humor outflow appeared satisfactory, the eye was pseudophakic, and successful ciliary sulcus implantation had previously been reported [6,7]. Subsequent examination of the explanted device suggested that intraluminal obstruction by uveal tissue was responsible for filtration failure. The timing of the obstruction suggests several possible mechanisms. Tissue from the posterior iris surface or ciliary body may have entered the PFM during implantation, causing partial obstruction while initially permitting aqueous humor flow. Alternatively, later migration of iris or other uveal tissue into the lumen, or direct obstruction caused by close apposition of adjacent tissue to the PFM opening, may have occurred. However, the precise timing and mechanism of the obstruction could not be determined. Nevertheless, this case demonstrates that visible intraoperative outflow does not exclude partial obstruction or ensure sustained postoperative patency. Intraoperative flushing might have cleared some of the tissue fragments, but whether this would have prevented postoperative obstruction remains uncertain.

The PFM was inserted with the bevel facing anteriorly (bevel up). Previous reports of ciliary sulcus implantation have described both anteriorly directed bevel-up placement [7] and posteriorly directed bevel-down placement [6]. In the present case, however, posterior placement of the PFM was unintentional. When intentionally performed, posterior implantation may offer advantages over conventional anterior chamber placement, including a greater distance from the corneal endothelium and a potentially lower risk of corneal endothelial cell loss. A posteriorly directed bevel may also reduce the likelihood of iris occlusion. Nevertheless, the optimal bevel orientation and its relationship to obstruction by adjacent uveal tissue remain uncertain, and this case cannot establish whether a different bevel orientation would have prevented the complication.

Corneal endothelial cell loss remains a concern following anterior chamber implantation of the PFM, and eyes with exfoliation glaucoma may be particularly vulnerable [8,9]. Intentional ciliary sulcus implantation has been reported to achieve IOP reduction with relatively stable CECD in an eye with a low preoperative endothelial cell count [7]. In the present case, CECD at three months after revision surgery was 2,376 cells/mm², approximately 8% lower than the value of 2,576 cells/mm² recorded at the initial examination. Because the reference measurement preceded microhook ab interno trabeculotomy and subsequent PFM implantation, this difference cannot be attributed specifically to PFM implantation or revision surgery. Continued monitoring is needed to assess subsequent endothelial changes.

Several limitations should be considered. First, the exact position of the original implant was not directly confirmed. The scleral tract was not deliberately created for ciliary sulcus placement, and the PFM may have followed a more anterior course along the posterior iris surface. In the previously reported technique for intentional posterior implantation, the needle was directed toward the ciliary sulcus at a steeper angle than that used for anterior chamber implantation [7]. Differences in trajectory may affect contact with adjacent uveal tissue and the likelihood of obstruction, although this remains speculative. Second, the presumed uveal origin of the obstructing material was based on stereomicroscopic appearance and the suspected implant position; its precise tissue of origin could not be established. Finally, this single case of unintended placement does not establish the efficacy or safety of intentional ciliary sulcus implantation.

## Conclusions

Inadvertent placement of the PFM posterior to the iris may lead to early postoperative obstruction by pigmented tissue consistent with uveal tissue, despite apparently satisfactory intraoperative aqueous humor outflow. This instructive case illustrates the importance of confirming the scleral tract trajectory and implant position, as initial flow alone does not ensure sustained tube patency. These findings highlight a potential complication of unintended placement but should not be generalized to the safety or efficacy of intentional ciliary sulcus implantation.
